# Suppression of Rice Blast by Bacterial Strains Isolated from Cultivated Soda Saline-Sodic Soils

**DOI:** 10.3390/ijerph17145248

**Published:** 2020-07-21

**Authors:** Yi Wei, Lanhui Li, Wenjun Hu, Huiyan Ju, Mingzhe Zhang, Qingming Qin, Shihong Zhang, Guihua Li

**Affiliations:** 1 College of Plant Sciences, Jilin University, Changchun 130062, China; wyziyu@163.com (Y.W.); lilh0205@163.com (L.L.); 18264901015@163.com (W.H.); jhuiyan@163.com (H.J.); mzzhang@jlu.edu.cn (M.Z.); qmqin@jlu.edu.cn (Q.Q.); zhang_sh@jlu.edu.cn (S.Z.); 2 Department of Plant Protection, Shenyang Agricultural University, Shenyang 110866, China

**Keywords:** *Magnaporthe oryzae*, rice blast disease, biological control, saline-sodic land, soil bacteria, antagonistic bacterial strain

## Abstract

Rice blast caused by *Magnaporthe oryzae* is one of the most serious rice diseases worldwide. Biological control is gaining popularity as a promising method for the control of this disease; however, more effective microbial strains with strong adaptability in rice fields need to be identified. Here, we report for the first time the successful identification of biocontrol bacterial strains from frozen soils of the soda saline-sodic land. We isolated 82 bacterial strains from rice fields in the western Songnen Plain of China, one of the three major soda saline soils in the world. Five of the isolated strains exhibited strong inhibition to *M. oryzae* growth. The potential strains were identified as *Bacillus safensis* JLS5, *Pseudomonas koreensis* JLS8, *Pseudomonas saponiphila* JLS10, *Stenotrophomonas rhizophila* JLS11 and *Bacillus tequilensis* JLS12, respectively, by 16s RNA gene sequence analysis. The antagonistic assay and the artificial inoculation tests showed that JLS5 and JLS12 could effectively inhibit conidial germination and pathogenicity of the rice blast fungus, both preventively and curatively. The suppression of pathogenicity was further confirmed by greenhouse experiments, showing the effectiveness of JLS5 and JLS12 as a potential biological control agents of *M. oryzae*. The potential application of these cold-tolerant strains for rice blast control in cold regions is discussed. Our data suggest that soda saline-sodic soils are a rich source for biocontrol strain isolation.

## 1. Introduction

Rice blast, caused by *Magnaporthe oryzae*, is the most serious disease of cultivated rice that results in 10–30% annual rice yield losses [1]. The blast fungus uses appressorium, a dome-shaped cell developed from germinated conidia, to mechanically breach the outer plant surface and infect host cells [2]. The main disease control method currently used is the application of chemical fungicides, which, albeit effective, pose severe threats to human health and environment as well as leading to drug resistance in fungi. Another common management strategy is to breed blast-resistant rice varieties. However, this method has proven to be ineffective for long-term control of the disease under field conditions [3]. Therefore, alternative disease management strategies that are both effective and environmentally friendly are highly desired.

Biological control of rice diseases using microorganisms has attracted increased attention in recent years owing to its superior features such as inhibition specificity, low toxicity, and lack of pathogen resistance [4,5,6]. Quite a few bacterial strains isolated from various environmental niches have shown a positive effect toward controlling *M. oryzae*. Some of the examples include the plant endophytic *Bacillus tequilensis* GYLH001 isolated from *Angelica dahurica* [7], and *Bacillus safensis* B21 from *Osmanthus fragrans* [8]. There are also bacteria isolated from the rhizosphere, including rhizobacteria [9], *Bacillus cereus* HS24 [10,11], and *Streptomyces* spp. [12] from the rice rhizosphere, and *Paenibacillus terrae* NK3-4 from the soybean rhizosphere [13]. In addition, some bacteria have been isolated from other environmental niches such as *Staphylococcus* sp. strain LZ16 from seawater [14], and *Streptomyces globisporus* JK-1 from a contaminated fungal culture [15].

Soils, especially those from saline-sodic land, are rich sources of bacteria that might exhibit antifungal effects. In this study, we isolated 82 bacterial strains from the rice fields in the western Songnen Plain of China, one of the three major soda saline soils in the world. Nine strains showed suppression ability against *M. oryzae* growth, five of which exhibited a strong inhibition effect. We further investigated in detail the antifungal effects of two strains, *Bacillus safensis* JLS5 and *Bacillus tequilensis* JLS12. Both strains could inhibit mycelial growth, conidial germination, and pathogenicity of *M. oryzae* effectively, suggesting their potential application as biological control agents against *M. oryzae*. This study showed that soda saline-sodic soils could be rich sources for the isolation of biocontrol strains.

## 2. Materials and Methods

### 2.1. Fungal Strains and Culture Conditions

Two Chinese *M. oryzae* field strains, Y34 [16,17] and JL0910 [18], were used in this study, which were cultured and maintained as reported previously [17].

### 2.2. Soil Sample Collection

Seven soil samples were collected from soda saline-alkali rice fields in the western Songnen Plain, located in Qian Gorlos Mongol Autonomous County, Songyuan, Jilin province of China. In brief, the surface-frozen soils were collected in December, and the samples were stored at 4 °C for ten days before the bacterial strain was isolated.

### 2.3. Isolation of Bacteria from Soil Samples

Five grams of soil in 50 mL sterile water was diluted to10^−6^, based on ten-fold gradient dilution. An aliquot of 100 μL each dilution was spread on Luria-Bertani (LB) plates and incubated at 28 °C for 24 h. Morphologically distinct colonies were purified and stored.

### 2.4. Screening of Antagonistic Strains against M. oryzae

The candidate biocontrol bacterial strains were cultured on PDA (potato, 200 g; glucose, 20 g; agar, 15 g; distilled water, 1 L), together with the target *M. oryzae* strain Y34 or JL0910 in the same plates, and the subsequent growth inhibitions of *M. oryzae* were observed.

### 2.5. Identification of Bacterial Strains by 16S rRNA Analysis

DNA used for PCR was isolated using the method reported previously [19]. The bacterial 16S rRNA genes were amplified using forward primer 27F (5′-AGAGTTTGATCCTGGCTCAG-3′) and reverse primer 1492R (5′-GGCTACCTTGTTACGACTT-3′) with the method reported previously [7]. The purified PCR products with a size of about 1.45 kb were sequenced. The sequences were deposited in the GenBank database, and the accession numbers were MT501806 (*B. safensis* JLS5), MT501807 (*P. koreensis* JLS8), MT501808 (*P. saponiphila* JLS10), MT501809 (*S. rhizophila* JLS11), and MT501810 (*B. tequilensis* JLS12). Sequence similarity was analyzed by the BLAST search program on the website of the National Center for Biotechnology Information (NCBI) (https://blast.ncbi.nlm.nih.gov/Blast.cgi). For phylogenetic analysis, published reference sequences for 16S rRNA genes were obtained from the NCBI database and aligned with the sequences obtained in this study by Clustal W within MEGA X [20]. The aligned sequences were tested for their phylogeny, and a neighbor-joining tree was generated using MEGA X to determine the relationships between the reference and unknown sequences.

### 2.6. Conidial Germination Assay

For *M. oryzae* conidial germination, three droplets (10 μL/each) of bacteria-treated and untreated conidial suspensions were loaded on a sterile glass slide (3 slides/treatment) and incubated at 28 °C in a moisture tray. The bacterial potato dextrose broth (PDB) cultures were diluted five-fold (about 10^8^ cells/mL) and used, with the same amount of PDB as control. The conidial germination was observed under microscopy, and the germination rate was recorded.

### 2.7. Pathogenicity Suppression Assays

Rice varieties Nipponbare and Jijing88, compatible with strains Y34 and JL0910 respectively, were used for the pathogenicity assay of *M. oryzae*. Plants were cultivated for two weeks after sowing, at the 3- to 4-leaf stage.

Two methods were used for rice inoculation. For the drop-inoculation approach [21], rice leaves of intact seedlings with soil-free roots were spread in a container, then were inoculated by dropping *M. oryzae* conidial suspension (1 × 10^5^ conidia/mL, 8 μL) on the leaf surface. Bacterial cultures were applied on the same point, before, simultaneously, or after *M. oryzae* inoculation. The spray-inoculation approach was used for disease control assays under greenhouse conditions. Rice seedlings were sprayed with *M. oryzae* conidial suspension (5 × 10^5^ conidia/mL in 0.03% Tween 20). Then, seedlings were placed in a humid and dark chamber for 24 h before being incubated further in a moist atmosphere with a 12-h light period. Bacterial cultures were applied to the rice seedlings before inoculation with *M. oryzae*. Symptoms were assessed visually at 7 DPI. Pathogenicity assay was performed as previously described [22]. Quantification of lesion size was performed with ImageJ software (https://imagej.nih.gov/ij/).

Detached leaves of the barley cultivar, Golden Promise, were used for pathogenicity suppression assays. A drop-inoculation approach similar to that mentioned above was used.

## 3. Results

### 3.1. Isolation and Screening of Antagonistic Bacterial Strains against M. oryzae from Soda Saline-Sodic Rice Fields

To identify antagonistic strains for rice blast disease, we collected seven soil samples from soda saline-sodic rice fields in the western Songnen Plain, in the Qian Gorlos Mongol Autonomous County, Jilin, China. The soil samples showed pH values of 7.94–8.51, CO_3_^2−^ levels of 0.001–0.002 g/kg, and HCO_3_^−^ levels of 0.03–0.04 g/kg (Table 1).

**Figure 1:**
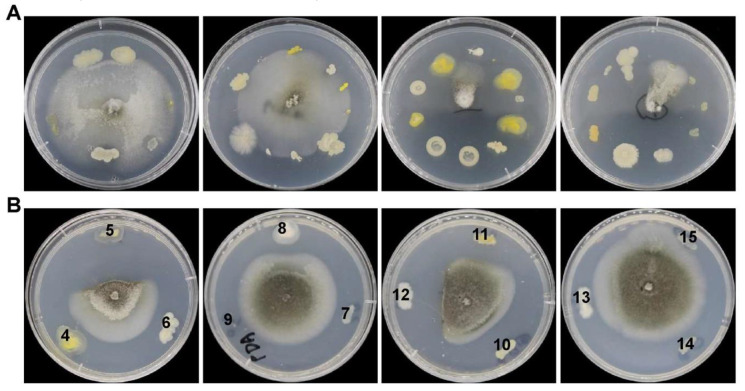
Screening of antagonistic bacterial strains isolated from soda saline-alkali cultivated soils against *M. oryzae*. (**A**,**B**) Primary screening (**A**) and secondary screening (**B**) are shown with typical PDA plates. Serial numbers 4 to 15 represent candidate bacterial antagonistic strains based on primary screening. The tested strain of *M. oryzae* is Y34.

A total of 82 strains were isolated from the seven soil samples. Next, we evaluated their inhibition efficiency against *M. oryzae* strain Y34 using a two-stage screening approach. For the primary screening, up to ten bacterial strains were inoculated onto the same PDA plate, surrounding *M. oryzae* located at the center of the plate (Figure 1A). The resultant candidate strains with antagonistic phenotypes were further evaluated by the secondary screening, with only three bacterial strains inoculated in each plate instead (Figure 1B). In the end, we obtained nine bacterial strains that showed significant inhibition to *M. oryzae* growth.

### 3.2. Molecular Identification of the Isolated Antagonistic Bacterial Strains

**Figure 2:**
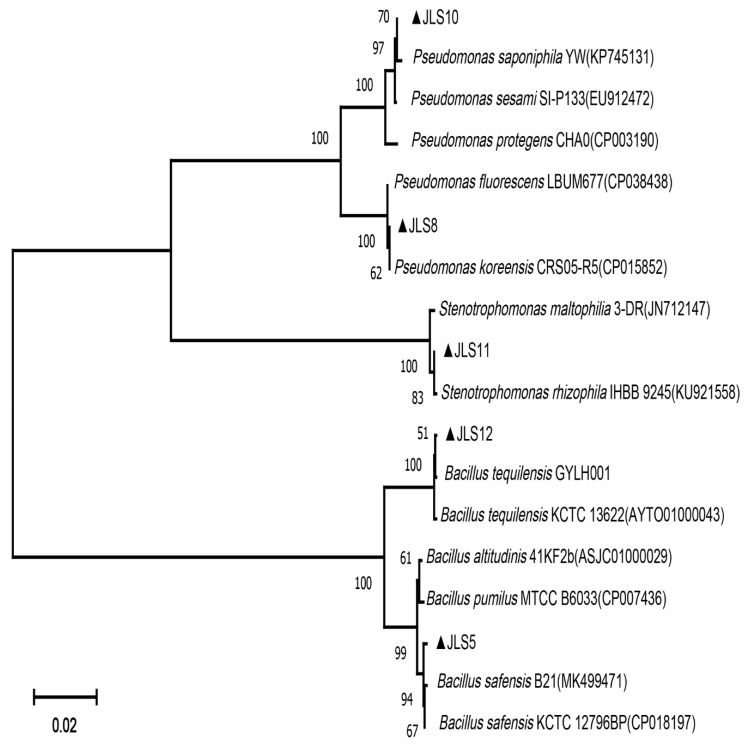
Neighbor-joining phylogenetic tree obtained from the 16S rRNA gene sequence data of the isolated antagonistic bacterial strains. Reference sequences obtained from the NCBI database are indicated by their accession numbers. Numbers at nodes are percentage bootstrap values based on 1000 replicates. The isolated antagonistic bacterial strains are marked with triangles, respectively, and their accession numbers were shown in the section of Materials and Methods.

Among the nine isolated antagonistic strains, five of them showed strong inhibition to *M. oryzae* growth (Table 2). A phylogenetic tree based on bacterial 16S rRNA gene sequences suggests that JLS5 and JLS12 belong to *Bacillus* (*Bacillus safensis* and *Bacillus tequilensis*, respectively); JLS8 and JLS10 belong to *Pseudomonas* (*Pseudomonas koreensis* and *Pseudomonas saponiphila*, respectively); JLS11 is *Stenotrophomonas rhizophila* (Figure 2 and Table 2). To our knowledge, this is the first report of *Pseudomonas saponiphila* and *Stenotrophomonas rhizophila* with antagonistic activities against *M. oryzae*.

### 3.3. Inhibitions of Mycelial Growth of M. Oryzae by the Isolated Antagonistic Bacterial Strains

**Figure 3:**
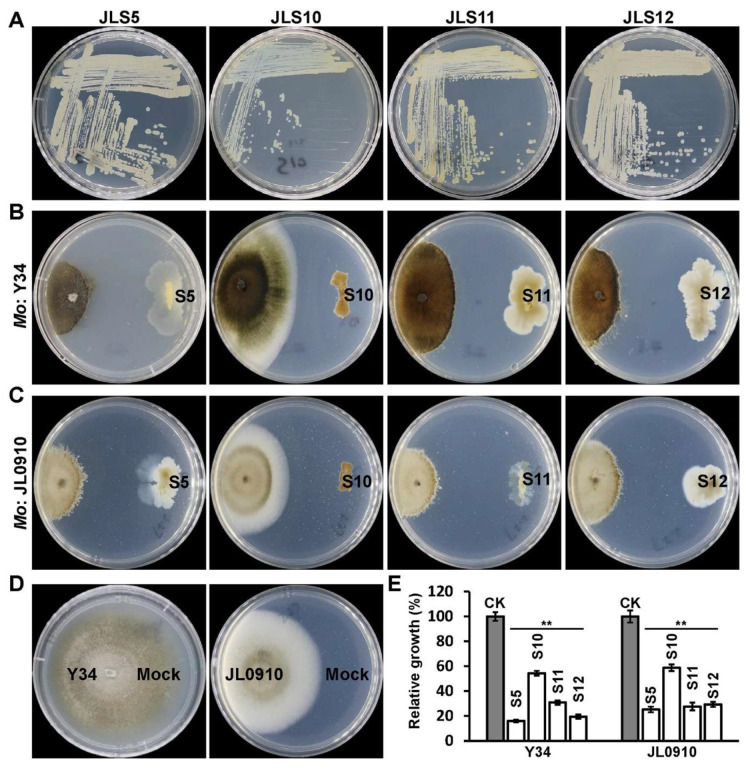
Inhibitions of *M. oryzae* growth by the isolated bacterial strains. (**A**) Colony morphology of four candidate biocontrol bacterial strains on LB plates. (**B**) Inhibitions of the colony growth of *M. oryzae* (*Mo*) strain Y34 by the four isolated bacterial strains. Representative photographs were taken at 10 days post-inoculation (DPI). S5, S10, S11, and S12 indicates bacterial strain JLS5, JLS10, JLS11, and JLS12, respectively. (**C**) Inhibition of the colony growth of *Mo* strain JL0910 by the four bacterial strains. Representative photographs were taken at 7 DPI. (**D**) Growth of the two *M. oryzae* strains, as control (CK). (**E**) Quantitative analysis of the growth inhibition caused by the indicated strains in (**B**,**C**). Data represent means ± standard deviation (SD) from at least three independent experiments. ** indicates statistical significance at *p* < 0.01.

The inhibition of *M. oryzae* growth by four isolated antagonistic bacterial strains was assayed further, by culturing only one bacterial strain facing *M. oryzae* on each PDA plate. Two *M. oryzae* wild-type strains, Y34 and JL0910 were used, respectively. The data showed that *B. safensis* JLS5 and *B. tequilensis* JLS12 could greatly inhibit the growth of *M. oryzae* Y34, with the growth rates reduced to about 20% of the control, i.e., showing 80% inhibition against Y34. The strain *S. rhizophila* JLS11 showed growth inhibition of about 70%. The suppression of *M. oryzae* growth by *P. saponiphila* JLS10 was relatively weak, with about 45% inhibition (Figure 3A,B,D,E). Similar results were also observed for the *M. oryzae* strain, JL0910 (Figure 3C–E). Our data showed that *B. safensis* JLS5 and *B. tequilensis* JLS12 exhibited the most effective biocontrol potential; therefore, both strains were studied further for the control of rice blast disease.

### 3.4. Bacterial Strains JLS5 and JLS12 Inhibit Conidial Germination of M. Oryzae

**Figure 4:**
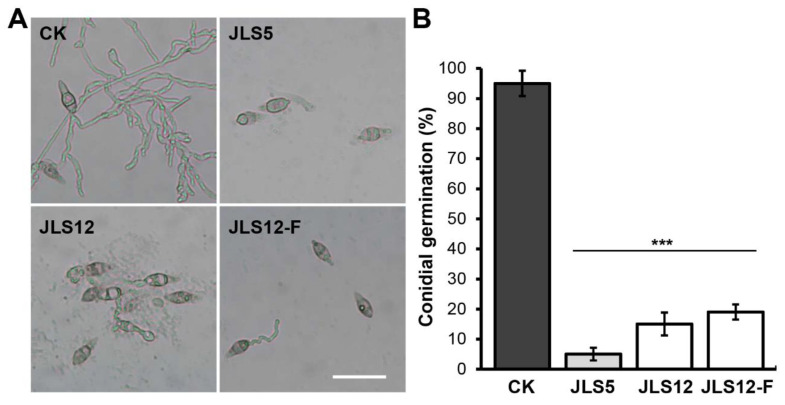
Inhibition of *M. oryzae* conidial germination by JLS5 or JLS12. (**A**) Conidial germination of *M. oryzae* Y34 is inhibited by the indicated bacterial strains, respectively. Scale bar indicates 50 μM. JLS12-F indicates the cell-free culture filtrates of JLS12. Representative photographs were taken after 12 h incubation. (**B**) Quantitative analysis of the conidial germination inhibition caused by the indicated strains in (**A**). Data represent means ± SD from at least three independent experiments. *** indicates statistical significance at *p* < 0.001.

Conidium is the primary source for *M. oryzae* spreading and infection in the field; inhibition of conidial germination is vital for rice blast disease control. Therefore, we evaluated the suppression efficiency of conidial germination by the isolated antagonistic bacterial strains JLS5 and JLS12, using five-fold dilutions of their PDB cultures (about 10^8^ cells/mL in the final content). The results indicated that both JLS5 and JLS12 could inhibit conidial germination of *M. oryzae* effectively. JLS5 treatment could inhibit 95% of conidial germination, showing only 5% of conidia germinated at 12 h post-incubation, compared to over 90% conidial germination in the control (Figure 4). JLS12 treatment could inhibit 85% of conidial germination. A similar result was also observed for the treatment with cell-free culture filtrates of JLS12, which inhibited about 81% of conidial germination (Figure 4). Notably, many bacteria-treated conidia suffered morphological changes or rupture. The resultant germinated conidia grew very slowly, with most of them showing very short germ tubes compared to that of the control (Figure 4).

### 3.5. Suppressions of M. Oryzae Pathogenicity on Host Leaves by the Isolated Antagonistic Bacterial Strains JLS5 and JLS12

**Figure 5:**
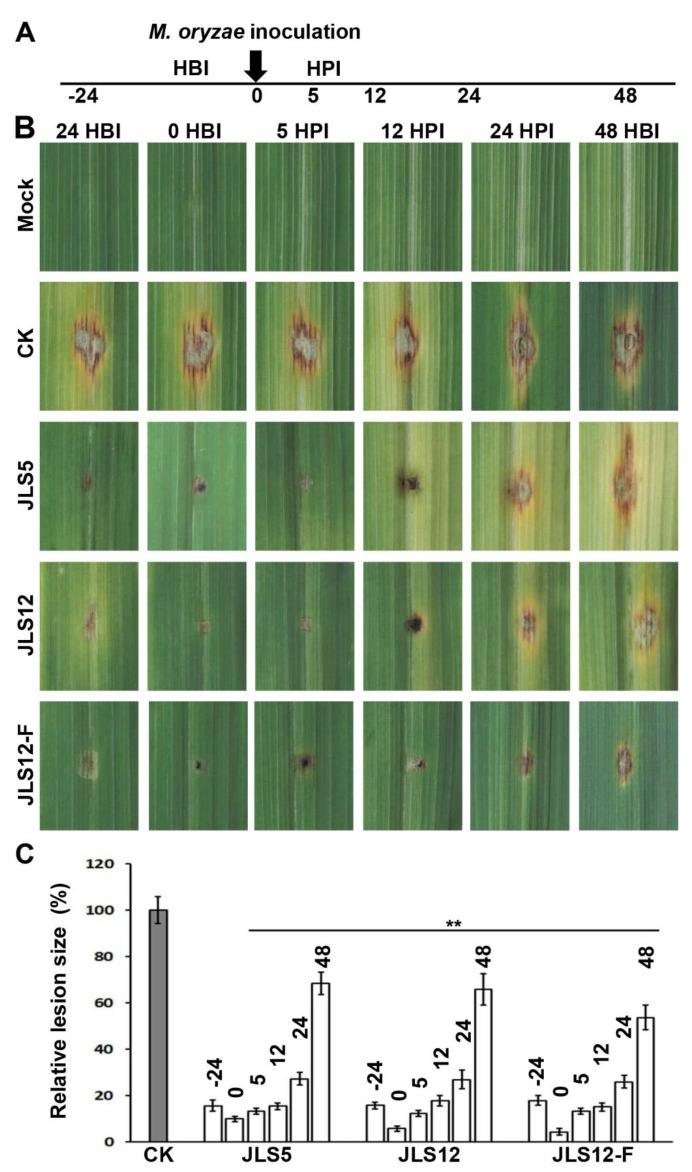
Suppression of *M. oryzae* pathogenicity on barley leaves by JLS5 or JLS12. (**A**) Schematic diagram of application time of biocontrol bacteria. According to the inoculation time of *M. oryzae*, biocontrol bacteria were applied at 24 h before inoculation (HBI), 0 (applied with *M. oryzae* conidia simultaneously), 5 h post inoculation (HPI), 12 HPI, 24 HPI and 48 HPI, respectively. (**B**) The pathogenicity of *M. oryzae* Y34 is inhibited by the indicated bacterial strains, respectively. *M. oryzae* were inoculated with a conidial drop on detached barley leaves. JLS12-F indicates the cell-free culture filtrates of JLS12. Representative photographs were taken at 7 days post-inoculation. (**C**) Quantitative analysis of the pathogenicity inhibition caused by the indicated strains in (**B**). −24, 0, 5, 12, 24, and 48 indicates 24 HBI, 0, 5 HPI, 12 HPI, 24 HPI, and 48 HPI, respectively. Data represent means ± SD from at least three independent experiments. ** indicates statistical significance at *p* < 0.01.

To evaluate the effects of JLS5 and JLS12 on *M. oryzae* pathogenicity, we inoculated their PDB cultures on detached barley leaves together with *M. oryzae*, and before or after *M. oryzae* inoculation at different time points (Figure 5A). Applications of bacterial culture before *M. oryzae* inoculation are to mimic preventive treatment, and applications after *M. oryzae* inoculation are for curative test. The application amount for each bacterial strain is a five-fold dilution of PDB culture (about 10^8^ cells/mL), the same as that involved in the conidial germination assay.

The results showed that both bacterial strains shared similar inhibition effects on *M. oryzae* pathogenicity, and all treatments could inhibit the pathogenicity significantly (Figure 5). The most effective control period for bacterial culture application is from 24 HBI to 12 HPI, during which the blast fungus has not penetrated into the host cells yet (Figure 5B). Applications of bacterial culture in this period could effectively inhibit the severity of rice blast, with suppression over 80%. Bacterial application at 24 HPI, when the blast fungus started to penetrate and colonize host cells, decreased the suppression ability to about 70%; when applying at 48 HPI, the inhibition was about 30% (Figure 5C). Similar results were observed when cell-free culture filtrates of JLS12 were used, and all applications from 24 HBI to 48 HPI could inhibit *M. oryzae* pathogenicity significantly (Figure 5B,C).

**Figure 6:**
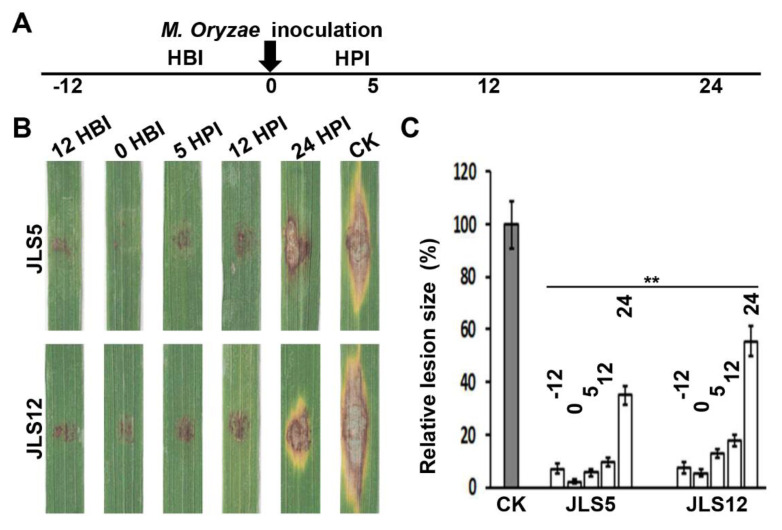
Suppression of *M. oryzae* pathogenicity on rice leaves by JLS5 or JLS12. (**A**) Schematic diagram of application time of biocontrol bacteria. According to the inoculation time of *M. oryzae*, biocontrol bacteria were applied at 12 h before inoculation (HBI), 0 (applied with *M. oryzae* conidia simultaneously), 5 h post-inoculation (HPI), 12 HPI and 24 HPI, respectively. (**B**) The pathogenicity of *M. oryzae* Y34 is inhibited by the indicated bacterial strains, respectively. *M. oryzae* were inoculated with a conidial drop on rice leaves. JLS12-F indicates the cell-free culture filtrates of JLS12. Representative photographs were taken at 7 days post-inoculation. (**C**) Quantitative analysis of the pathogenicity inhibition caused by the indicated strains in (**B**). −12, 0, 5, 12 and 24 indicates 12 HBI, 0, 5 HPI, 12 HPI and 24 HPI, respectively. Data represent means ± SD from at least three independent experiments. ** indicates statistical significance at *p* < 0.01.

To confirm the pathogenicity inhibition observed on barley, we did similar tests on rice with the drop-inoculation method (Figure 6A), and similar results were observed. Applications of JLS5 or JLS12, during the period from 12 HBI to 12 HPI, inhibited *M. oryzae* pathogenicity effectively, with the inhibition over 90% and 80%, respectively (Figure 6B,C). Even application at 24 HPI, the inhibition remained at 65% and 45%, respectively (Figure 6B,C).

These tests indicated that both JLS5 and JLS12 display excellent preventive effects on the pathogenicity of *M. oryzae*. Although a good curative effect can be obtained when applied at the early infectious stage of the rice blast fungus, the control effect of the bacterial strains decreased significantly. Therefore, when using these biocontrol bacteria to control rice blast disease, prevention should be the priority.

### 3.6. Control of Rice Blast Disease on Rice Seedlings by the Isolated Antagonistic Bacterial Strains JLS5 and JLS12 in Greenhouse

**Figure 7:**
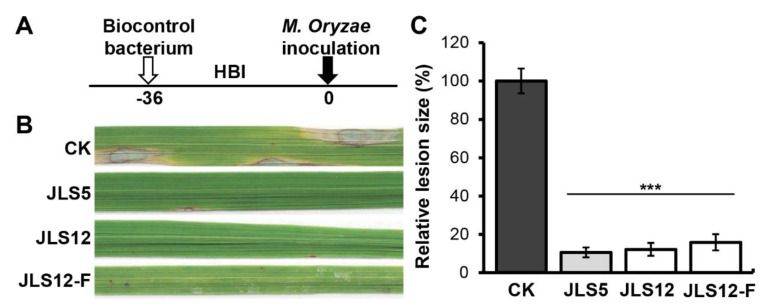
Control of rice blast by JLS5 or JLS12 under greenhouse conditions. (**A**) Schematic diagram of the treatment time of biocontrol bacteria. Bacterial strains were applied at 36 h before inoculation (HBI) of *M. oryzae*. (**B**) The pathogenicity of *M. oryzae* Y34 is inhibited by the indicated bacterial strains, respectively. *M. oryzae* were inoculated by conidial spraying on rice seedlings in the greenhouse. JLS12-F indicates the cell-free culture filtrates of JLS12. Representative photographs were taken at 7 days post-inoculation. (**C**) Quantitative analysis of the pathogenicity inhibition caused by the indicated strains in (**B**). Data represent means ± SD from at least three independent experiments. *** indicates statistical significance at *p* < 0.001.

Since JLS5 and JLS12 exhibited strong preventive suppressions against *M. oryzae*, we subsequently evaluated their control effects on rice blast disease in the greenhouse with a preventive approach. Rice seedlings were sprayed with five-fold dilutions of bacterial PDB cultures with 10^8^ cells/mL in content at 36 h before inoculation of *M. oryzae* (Figure 7A). The results showed that rice blast disease was controlled effectively by the preventive application of JLS5 and JLS12, respectively. Only rare tiny lesions appeared on rice leaves, which were pre-applied with bacterial cultures (Figure 7B); over 85% of inhibition was observed based on lesion size calculation (Figure 7C). A similar result was found when 20% of cell-free culture filtrates of JLS12 were used (Figure 7B,C). These greenhouse tests indicated that the PDB cultures of JLS5 and JLS12 could be used for effective control of rice blast disease.

## 4. Discussion

In recent years, quite a few potential biocontrol strains for rice blast suppression have been isolated from plant rhizosphere soils [9,10,11,12,13]; however, none of them were collected from saline-sodic fields. In this study, we have isolated 82 bacterial strains from the soda saline-sodic fields in the western Songnen Plain of China, with several of the strains showing potentials as biological control agents of *M. oryzae*. Saline-sodic soil is a type of degraded land, and approximately 1.1 × 10^9^ hectares of soil worldwide are saline-affected [23]. The western Songnen Plain is one of the three major regions with saline-sodic soils in the world containing over 3.0 × 10^6^ hectares of soda saline-sodic lands [24]. Our study indicates that these lands are rich sources for biocontrol strain isolation to inhibit rice blast or other plant fungal diseases. To our knowledge, this is the first report of biocontrol strain isolation from soda saline-sodic fields.

We have identified five bacterial strains with significant inhibition against *M. oryzae* in this study, which are *B. safensis* JLS5, *P. koreensis* JLS8, *P. saponiphila* JLS10, *S. rhizophila* JLS11 and *B. tequilensis* JLS12. To our knowledge, this is the first report showing that *P. saponiphila* and *S. rhizophila* display antagonistic activities against *M. oryzae*; their applications in controlling rice blast and the associated mechanisms should be studied further. The five antagonistic bacterial strains isolated in this study do not show adverse effects on rice growth and development. Among them, *B. safensis* JLS5 and *B. tequilensis* JLS12 exhibited strong suppression of mycelial growth, conidial germination, and pathogenicity of the rice blast fungus. This inhibition was much more effective when the bacterial agents were applied before *M. oryzae* penetration. The results suggest that, when using these strains to manage rice blast disease, we should apply them before symptoms appear, which is also the common feature of biological control. Recently, we have been trying to apply *B. tequilensis* JLS12 to manage rice blast disease in the field with preventive strategy. The control effect of the first-stage application appeared encouraging, which will be confirmed further in the subsequent growing seasons.

It should be pointed out that two related strains, *B. safensis* B21 [8] and *B. tequilensis* GYLH001 [7], have been reported previously as potential biological control agents of *M. oryzae*. Both strains were also isolated from China; however, in contrast to our strains (*B. safensis* JLS5 and *B. tequilensis* JLS12) that are isolated in cold winter from soda saline-sodic soils located in Northeast China, both of the other reported strains GYLH001 and B21 were plant endophytic bacteria isolated in the growing season from *Angelica dahurica* (a traditional Chinese medicine) and *Osmanthus fragrans* located in warmer regions of China, respectively. Therefore, we suspect that these strains would show distinct features that are representative of their ecological niches. The difference in biological control mechanisms between them needs to be studied further. Since the bacterial strains reported here were isolated from extreme cold frozen soil (below −20 °C) in winter, they are suitable for protecting rice production in cold regions (e.g., Northeast China). In addition to being effective agents for rice blast management in growing seasons, these cold-adapted bacterial strains have the potential to inhibit *M. oryzae* (conidia and/or mycelia) survival in diseased rice tissues or soils during the non-growing season, and to attenuate their primary infection in the next growing season. This non-growing season management of rice blast is usually ignored in the current popular biological control strategies. The promising application of inhibiting the survival of the blast fungus in non-growing seasons is worthy of a comprehensive study in the near future.

In summary, five bacterial strains with significant inhibition against *M. oryzae* growth were isolated from soda saline-sodic soils in this study. Two of them showed strong suppression of conidial germination and pathogenicity of *M. oryzae*, indicating the potential application of both strains as biological control agents of rice blast. Our data suggests that soda saline-sodic soils can be rich sources for biocontrol strain isolation. 

## 5. Conclusions

Our study is the first report to successfully isolate and identify biocontrol bacterial strains from soda saline-sodic land for the management of rice blast disease. A total of 82 bacterial strains were isolated, and five of them exhibited strong inhibition of mycelial growth of *M. oryzae*. The strains were identified as *B. safensis* JLS5, *P. koreensis* JLS8, *P. saponiphila* JLS10, *S. rhizophila* JLS11, and *B. tequilensis* JLS12. PDB cultures of JLS5 or JLS12 exhibited strong inhibition effects on conidial germination and pathogenicity of the rice blast fungus, both preventively and curatively. The suppressions of pathogenicity were further confirmed by greenhouse experiments, indicating that both strains have potential applications for biological control of *M. oryzae*. This study suggests that soda saline-sodic soils can be rich sources for biocontrol strain isolation.

## Figures and Tables

**Table 1:** Soil sample parameters and the number of isolated bacterial strains.

Soil Sample	pH	Collection Location		Altitude (m)	Numbers of Bacteria	
		North Latitude	East Longitude		Total	Antagonistic ^1^
#1	8.03	45°01′06″	124°36′10″	130	16	2
#2	8.08	45°01′06″	124°36′10″	130	11	0
#3	7.94	45°01′06″	124°36′10″	130	11	1
#4	8.51	44°57′19″	124°38′14″	140	11	0
#5	8.11	44°59′40″	124°40′54″	130	11	0
#6	8.00	44°59′40″	124°42′47″	140	11	2
#7	8.19	45°00′00″	124°42′27″	130	11	4

^1^ Antagonistic strains screened with suppression ability against *M. oryzae* growth.

**Table 2:** Isolated bacterial strains with strong inhibition to *M. oryzae* growth.

Strain	Inhibition Effect on *M. oryzae* Growth ^1^	Identification	Soil Sample
JLS5	+++++++	*Bacillus safensis*	#6
JLS8	+	*Pseudomonas koreensis*	#7
JLS10	+++	*Pseudomonas saponiphila*	#7
JLS11	+++++	*Stenotrophomonas rhizophila*	#7
JLS12	+++++++	*Bacillus tequilensis*	#7

^1^ Determined by plate growth inhibition. The more the number of “+” the higher the degree of inhibition.
